# Critical illness-related corticosteroid insufficiency (CIRCI) in paediatric patients: a diagnostic and therapeutic challenge

**DOI:** 10.1186/s13052-024-01616-x

**Published:** 2024-03-11

**Authors:** Letteria Anna Morabito, Domenico Corica, Giorgia Pepe, Alessandra Li Pomi, Tommaso Aversa, Malgorzata Gabriela Wasniewska

**Affiliations:** 1 Pediatric Unit, Maternal Infant Department, “Gaetano Martino” University Hospital, Via Consolare Valeria, 98122 Messina, Italy; 2 Department of Human Pathology in Adulthood and Childhood, University of Messina, Messina, Italy

**Keywords:** Adrenal insufficiency, Paediatric critical care, Critical illness related corticosteroid insufficiency

## Abstract

Critical illness-related corticosteroid insufficiency or CIRCI is characterized by acute and life-threatening disfunction of hypothalamic–pituitary–adrenal (HPA) axis observed among intensive care unit- staying patients.

It is associated with increased circulating levels of biological markers of inflammation and coagulation, morbidity, length of ICU stay, and mortality.

Several mechanisms are involved in CIRCI pathogenesis: reduced CRH-stimulated ACTH release, peripheral resistance to glucocorticoids, altered cortisol synthesis, impaired cortisol-free fraction and bioavailability.

Diagnostic and therapeutic management of this condition in children is still debated, probably because of the lack of agreement among intensive care specialists and endocrinologists regarding diagnostic criteria and prevalence of CIRCI in paediatric age.

In the present narrative review, we focused on definition of CIRCI in paediatric age and we advise on how to diagnose and treat this poorly understood condition, based on current literature data.

## Introduction

Critical illness-related corticosteroid insufficiency (CIRCI) is a condition characterized by reduced adrenal steroid production due to dysfunction at any point in the hypothalamic–pituitary–adrenal (HPA) axis or due to tissue resistance to glucocorticoids in the absence of anatomic lesions at any point. It is observed mainly in intensive care unit (ICU)-staying patients, affected by severe physical stress, due to sepsis, major trauma, intracranial bleeding, cardiac surgery, and systemic inflammation syndromes (e.g., SIRS, hemophagocytosis).

The first definition of CIRCI was made in 2008 by a multispecialty task force of the Society of Critical Care Medicine (SCCM) and the European Society of Intensive Care Medicine (ESICM) [1].

Both adult and paediatric critical patients could develop CIRCI because of reduced cortisol production and/or resistance to cortisol action during critical illnesses.

The association between insufficient glucocorticoid activity and excessive production of proinflammatory mediators [2] could lead to an exaggerated and protracted inflammatory response with organ failure, prolonged ICU stay and higher ICU mortality [3].

The exact prevalence of CIRCI in ICU patients is poorly understood. More recent literature has shown an incidence ranging widely from 30 to 70%.

The reason for this wide variability depends on several factors, including differences among study populations, type and severity of patient illness, differences among treatment regimens and study inclusion criteria of the population being studied [4].

Data about CIRCI in paediatric ICU patients are also very poor because of the lack of agreement among intensive care specialists and endocrinologists regarding diagnostic criteria and prevalence of this condition in paediatric age [5] (Table 1).

**Table 1:** Summary of recent literature studies about adrenal insufficiency in critically ill children

First Author name (year)	Critical diseases associated with adrenal insufficiency or CIRCI	Pediatric population features (number, gender, median age)	ACTH test type (high/ low dose)	Diagnostic criteria for adrenal insufficiency	Clinical complications of unrecognized adrenal insufficiency
Pizarro C (2005) [5]	Septic shock	57 patients (23 M, 34 F) Median age: 27 months	High dose ACTH test (250 μg Synacthen)	Cortisol response after ACTH administration (delta cortisol) < 9 μg/dL Absolute adrenal insufficiency: baseline cortisol < 20 μg/dL Relative adrenal insufficiency: baseline cortisol > 20 μg/dL	- Catecholamine-resistant shock - Worsening of multiple organ failure - High mortality
Menon K (2010) [6]	Major trauma, sepsis, cardiac surgery, general surgery	381 patients (198 M, 183 F); Median age: 4.01 years	Low dose ACTH test (1 μg Synacthen on day 1) ** + ** High dose ACTH test (1 μg + 250 μg Synacthen on day 2)	Cortisol response after ACTH administration < 9 μg/dL in both tests	- Need for a greater number of catecholamines (*P* < 0.001), - Greater number of days on catecholamines (*P* = 0.002), and more fluid boluses
Hebbar A (2011) [7]	Systemic inflammatory response syndrome (SIRS) secondary to septic shock, Trauma/intracranial Bleeding, Seizures, Aspiration pneumonitis, Cardiopulmonary arrest, Pulmonary hypertension, Hemophagocytosis, Cytarabine toxicity, Systemic lupus Erythematosus, Alveolar dysplasia	78 patients (45 M, 33 F) Median age: 84 months	Low dose ACTH test (1 μg Synacthen)	Basal cortisol level < 18 mg/dL Relative adrenal insufficiency: difference between baseline cortisol and cortisol levels following low-dose ACTH stimulation of < 9 μg/dL	- Hypotension following fluid resuscitation - Vasopressor-dependent shock
Demiral M (2019) [8]	Various critical illnesses (infections, neurological diseases, cardiac diseases, intoxication, sepsis, renal insufficiency, diabetic ketoacidosis, and autoimmune hemolytic anemia)	100 patients (45 F, 55 M) Median age: 7.0 years	Low dose ACTH test (1 μg Synacthen)	Peak cortisol levels < 18 μg/dL or delta cortisol levels < 9 μg/dL following ACTH stimulation	- High underline disease severity - High mortality risk

*M* Males, *F* Females

## Methods

The authors focused their search on the diagnostic criteria and therapeutic management of CIRCI in paediatric age in the context of existing literature data.

The authors performed a literature search in PubMed and EMBASE, using selected key words (‘Critical illness- related corticosteroid insufficiency OR CIRCI’) AND (‘children OR newborns’) AND (‘diagnosis OR definition’) AND (‘adrenal Insufficiency OR adrenal failure’). Besides the automated search, a manual search for additional relevant publications was carried out in the bibliographies of the papers automatically identified.

All authors independently identified the most relevant papers published in English in the past 18 years, including original papers, clinical trials, and reviews. Case reports, series, and letters were excluded.

The contributions were critically reviewed and collected by all the authors, all of whom approved the final version.

## Pathophysiology of the adrenal axis during critical illnesses

Critical illness represents a severe form of physical stress. The activation of the stress response is crucial for general adaptation to illness and stress and contributes to the maintenance of cellular and organ homeostasis [9]. It is mediated by two important systems: the HPA axis and the sympatho-adrenal system (SAS), which are functionally related.

Activation of the HPA axis is one of the most important events in the stress “fight or flight” response.

It results in increased secretion of corticotropin-releasing hormone (CRH) and arginine vasopressin from the paraventricular nucleus of the hypothalamus.

CRH stimulates the production of ACTH (or corticotropin) by the anterior pituitary, causing the zona fasciculate of the adrenal cortex to produce more glucocorticoids (cortisol in humans).

Arginine vasopressin acts synergistically with CRH in corticotropin secretion.

The increase in cortisol production results in multiple systemic effects.

This hormone plays a key role in controlling inflammation, and providing essential metabolic substrates, for fluid retention and activation of the cardiovascular system to increase blood pressure and cardiac output [10].

Cortisol exerts its effects following uptake from the circulation by binding to intracellular glucocorticoid receptors. The cortisol – glucocorticoid receptor complex moves to the nucleus, where it binds as a homodimer to DNA sequences called glucocorticoid-responsive elements located in the promoter regions of target genes, which then activate or repress transcription of the associated genes.

A recent task team has described three major pathophysiological events in CIRCI: dysregulation of the hypothalamic-pituitary-axis, altered cortisol metabolism, and tissue corticosteroid resistance [1].

### Dysregulation of the hypothalamic-pituitary-axis

CRH secretion during stress could be modulated by several factors, including adrenergic agonists, catecholamines, opioids, serotonin, angiotensin II, vasoactive intestinal peptide (VIP) and a wide number of inflammatory cytokines: interleukin (IL)-1, IL-2, IL-6, and tumor necrosis factor-α (TNF-α) [11].

TNF-alpha impairs CRH-stimulated ACTH release and reduces adrenal cortisol synthesis by inhibiting the stimulatory actions of ACTH and angiotensin II on adrenal cells [12, 13].

Interleukin-1 blocks glucocorticoid receptor translocation and transcription [14].

**Fig. 1:**
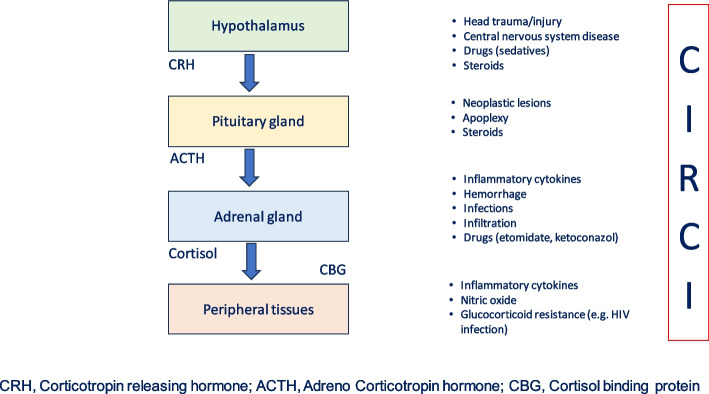
Factors influencing HPA axis function in paediatric patients with CIRCI

These molecules influence HPA axis function during severe illnesses, leading to inadequate production of cortisol (Fig. 1).

### Tissue corticosteroid resistance

Peripheral resistance to glucocorticoids may also occur in critically ill children as a consequence of abnormalities in glucocorticoid receptors or increased tissue conversion of cortisol to inactive cortisone.

Other studies also showed that high cortisol levels during critical illness could be a consequence of decreased metabolism and clearance of cortisol rather than increased production [15, 16].

### Altered cortisol metabolism

Decreased production of cortisol and/or ACTH is particularly common in patients with severe sepsis and septic shock.

In CIRCI patients, cortisol synthesis could be influenced by substrate deficiency, such as LDL cholesterol, whose levels often fall in patients with many acute illnesses, including sepsis and burns [17, 18].

During critical illness, the cortisol-free fraction and bioavailability increase because of the reduction in plasma albumin and corticosteroid-binding globulin, downregulation of hepatic glucocorticoid receptors, altered protein binding, increased volume of distribution, and altered 11β-hydroxysteroid dehydrogenase activity [19].

A first and immediate peripheral driver of increased systemic cortisol availability is the pronounced decrease in the levels of cortisol plasma binding proteins and the reduction of the cortisol-binding affinity [20, 21].

The second is a uniformly suppressed enzymatic cortisol breakdown in the kidney and liver [10].

## CIRCI in paediatric age: a challenging diagnosis

The exact prevalence of CIRCI in children is uncertain because the diagnostic criteria are unclear.

The term CIRCI has been used since 2008 to describe disruption of the hypothalamic–pituitary–adrenal (HPA) axis during critical illness [22].

However, the incidence, importance, and therapeutic approach to adrenal insufficiency in critically ill children are still poorly understood [23].

**Fig. 2:**
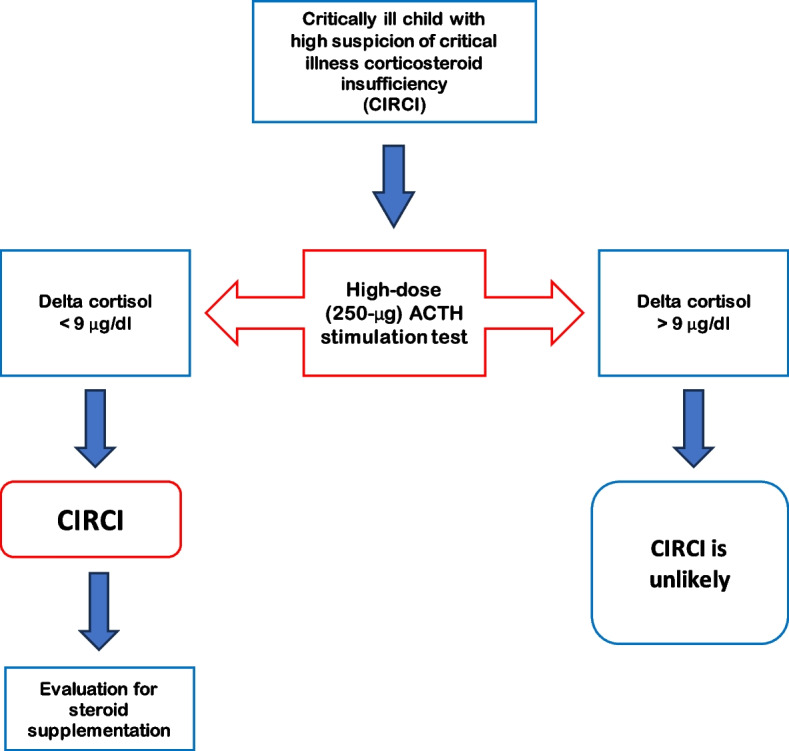
Diagnostic approach for CIRCI in paediatric patients

The term CIRCI should not be used to describe impaired function of the HPA axis already present prior to the onset of a critical illness, such as preexisting (undiagnosed/untreated/latent) intrinsic disease affecting the HPA axis (e.g., Addison’s disease), which led to a full-blown adrenal crisis triggered by stress conditions (Fig. 2) [24].

Clinically apparent adrenal insufficiency is usually rare in critically ill patients [25].

Hemodynamic instability and vasopressor treatment dependancy are strong indicators of CIRCI [26].

Central nervous system dysfunction is common and is frequently compounded by the underlying disease. In addition, CIRCI should be considered in critically ill patients with unexplained fever, persistent hypoxia, or difficult weaning from mechanical ventilation [27]. Laboratory assessment may reveal eosinophilia and hypoglycaemia during stress or fasting. Hyponatremia and hyperkalaemia are uncommon [9].

Literature data about CIRCI in children showed that the presence of adrenal insufficiency is associated with a refractory shock state and increased mortality rate in septic shock patients [28].

The diagnosis of CIRCI should also be considered in patients who have an exaggerated or uncontrolled proinflammatory response. This would include patients with ARDS, burns, pancreatitis and liver failure, who are at an increased risk of developing CIRCI [9].

Moreover, CIRCI should be suspected even in children hospitalized in the cardiac critical care unit, especially in cases of hypotension unresponsive to vasopressors or inotropes [29].

### Diagnostic controversies: ACTH stimulation test vs random cortisolemia

Diagnostic criteria for CIRCI in children are still debated. Moreover, there is no consensus on which method (baseline or stimulated plasma cortisol or increment in cortisol levels) should be used to diagnose adrenal insufficiency [30].

In recent years, the concepts of “relative” and “absolute” adrenal insufficiency (RAI and AAI, respectively) have been introduced to classify the failure of the adrenal gland to produce sufficient amounts of steroid hormones, primarily cortisol, to meet the peripheral requirements in critical patients.

Baseline cortisol levels and delta cortisol values (defined as the change in baseline cortisol levels 60 min after cosyntropin bolus administration) have both been used as biochemical indicators of adrenal insufficiency.

RAI was characterized by baseline cortisol levels < 20 μg/dL and an increment < 9 μg/dL [28].

AAI has been defined by the presence of baseline cortisol levels < 18 μg/dL [31].

Many threshold concentrations have been proposed to define the “appropriate” incremental response of cortisol after a cosyntropin bolus [32].

The 2008 guidelines by the American College of Critical Care Medicine suggested that the diagnosis of CIRCI is best made by a delta total serum cortisol of < 9 μg/dL after IV cosyntropin (250 μg) administration or a random total cortisol of < 10 μg/dL [33].

The latest guidelines, made in 2017 and valid for both critically ill adults and children, clarify some doubts about biochemical criteria for the diagnosis of CIRCI [1].

The high-dose (250 μg) ACTH stimulation test remains the best diagnostic test for CIRCI. It is superior to other existing methods to establish the diagnosis of adrenal insufficiency [34], easy to perform and safe. The patient’s increase in plasma total cortisol of ≤ 9 μg/dL after ACTH stimulation could be interpreted as CIRCI (Fig. 2) [1].

Other methods, such as random plasma cortisol measurement, are not recommended to make the diagnosis of adrenal insufficiency.

Additionally, salivary cortisol level dosages are not useful for this purpose, because they provide information only on free cortisol levels and may be influenced by several confounding factors, such as sex, age, time and site of sampling, and saliva volume.

No evidence exists about the use of serum free cortisol or ACTH levels to establish the presence of adrenal dysfunction in critically ill patients.

Because of the broad spectrum of abnormalities that may cause CIRCI (altered cortisol synthesis or metabolism, tissue resistance to cortisol), it is not reasonable to ascertain that a single test could reliably perform its diagnosis.

The major criticism of the 250 mg ACTH stimulation test is the use of a pharmacologic dosage that results in supraphysiologic stimulation, especially in small children, thus potentially inducing a cortisol response in patients with inadequate adrenal reserve [35].

The reason for this important bias could be found in the lack of randomized trials that compared other methods to diagnose CIRCI.

## Management

Literature data about the therapeutic management of children with CIRCI are lacking, and the question about what type of intervention should be appropriated is still debated.

Current guidelines [1] recommend the use of hydrocortisone in patients with septic shock that is not responsive to fluid and moderate- to high-dose vasopressor therapy (> 0.1 μg/kg/min of norepinephrine or equivalent) and/or in those with early severe acute respiratory distress syndrome (PaO2/FiO2 of < 200 and within 14 days of onset).

The beneficial effect of hydrocortisone in these patients has not been well ascertained, so the risks and benefits of this approach continue to be explored, especially in the paediatric field.

Glucocorticoids are effective in restoring cardiovascular homeostasis in sepsis through genomic and nongenomic effects [36], so they appear to be very useful in the management of adrenal insufficiency.

Several factors could influence the response to glucocorticoid administration in CIRCI patients: the type of underlying disease and the complex pathogenesis of this condition, involving different mechanisms, such as reduced steroid clearance and corticosteroid resistance.

Among adult patients, the treatment protocol involved the administration of 50 mg intravenous hydrocortisone every 6 h or a bolus of 100 mg followed by continuous infusion at 10 mg/h [26]. No indications are provided about hydrocortisone dosage in paediatric patients with CIRCI.

Treatment with a stress dose of hydrocortisone may be started early and without the need for dynamic testing if the random cortisol level is < 276 nmol/L (10 μg/dL). If random cortisol levels are between 276 and 938 nmol/L (10–34 μg/dL) and the 250 μg ACTH test reveals a delta cortisol < 248 nmol/L (9 μg/dL), the diagnosis of CIRCI is made, and glucocorticoid treatment is indicated.

Additionally, the question about the duration of steroid supplementation is still debated. A recent work, by Arcellana et al. [37] indicated that 200 mg/day hydrocortisone should be given for at least 72 h and up to 7 days, to have a significant benefit [1, 38], but no univocal indication is provided about this topic.

### Patients with sepsis

Hydrocortisone supplementation in septic patients has been extensively studied in the last 20 years.

Several studies have shown that sepsis induces changes in HPA axis activity, cortisol metabolism and peripheral tissue resistance to glucocorticoids, so the risk of developing CIRCI is very high in this population.

It has been observed that the presence of adrenal failure in patients with a septic state (including both sepsis and septic shock) is associated with increased mortality and a long duration of intensive care unit stay. It also seems that the need for inotropic support and high doses of inotropic drugs might be considered as an indicator of adrenal insufficiency in ICU patients [8].

The 2019 Cochrane review about the use of corticosteroids for treating sepsis in children and adults confirms that patients with septic shock may obtain a survival benefit from corticosteroid treatment, but it could not be the same for those with CIRCI [7].

Moreover, the analysis of trials including patients with CIRCI does not show a significant reduction in the risk of death at 28 days [1].

The inconsistency of data derived from the few clinical studies on this topic has probably affected the results shown by the Cochrane review, so the examination of the effects of glucocorticoid supplementation in critically ill children is becoming increasingly necessary.

Hebbar et al. examined corticosteroid supplementation for patients with SIRS with or without sepsis.

Children with hypotension, unresponsive to fluid resuscitation, and on IV vasopressor infusions have been treated with a loading dose of IV hydrocortisone (100 mg/m^2^) followed by 25 mg/m^2^ per dose given every 6 h for 7 days without any taper [39] (Table 2).

**Table 2:** Clinical and biochemical features of suspected CIRCI at various age and suggested management approach

**Patients at high risk of Adrenal insufficiency**	**Clinical features of Adrenal insufficiency and suspected CIRCI**	**Need for ACTH test in order to perform CIRCI diagnosis**	**Biochemical criteria for adrenal insufficiency**	**Therapeutic approach**
**Neonatal age** **(No clear definition/ evidence about CIRCI)**				
Asphyxiated newborns Preterm newborns	Hemodynamic instability Increasing need for catecholamine administration Increasing risk for heart dysfunction	Yes, low dose ACTH test	- Random cortisol levels < 15 mcg/dl - Total stimulated cortisol levels after ACTH administration < 17 mcg/dl	Hydrocortisone 50 mg/m2/day or 1 mg/kg every 8 h *Careful evaluation of patients susceptible to hydrocortisone treatment because of the risk of neurodevelopmental and gastrointestinal adverse effect*
**Pediatric age**				
Septic shock	Fluid unresponsive shock, vasopressor-dependent shock, hypoglycemia	Yes, high dose ACTH test (250 mcg)	Stimulated cortisol increment < 9 mg/dl over baseline	Hydrocortisone bolus of 100 mg/m2 followed by 25 mg/m2/dose every 6 h without any taper, especially until laboratory results *Consider also treatment with 50 mg/m2/day of hydrocortisone as alternative treatment* Discontinuation of treatment if criteria are not met
Acute respiratory distress syndrome (ARDS)	Shock, strong dependence/ difficult weaning from mechanical ventilation	Not clarified	Not clarified	Corticosteroids are not recommended as routine therapy *Consider methylprednisolone at a dose of 1 mg/kg/day if ARDS and a PaO2/FiO2* < *200 within the first 6 days of illness with slow tapering*
Meningococcal disease	Fluid unresponsive shock, vasopressor-dependent shock, hypoglycemia	Yes, high dose ACTH test (250 mcg)	Stimulated cortisol increment < 9 mg/dl over baseline	Early Hydrocortisone bolus of 100 mg/m2 followed by 25 mg/m2/dose every 6 h without any taper, especially until laboratory results
Major trauma Severe burns	Uncontrolled inflammation, vasopressor dependency	Not clarified	Not clarified	Steroid supplementation is not recommended because of the absence of improvement in short-term mortality

Early initiation of steroid therapy is particularly useful in some patients, even in the presence of sepsis without shock [40, 41].

They are represented by children with purpura fulminans and Waterhouse-Friedrichsen syndrome, children who previously received steroid therapies for chronic illness, and those with pituitary or adrenal abnormalities. The risk of adrenal failure is very high in these patients, and prompt initiation of hydrocortisone treatment could prevent acute adrenal crisis.

### Newborn patients

Several conditions are associated with neonatal adrenal insufficiency, but no clear definition of CIRCI in neonatal age has already been provided.

Early diagnosis of adrenal insufficiency seems to be important, especially in asphyxiated neonates with hemodynamic instability because of the well-known positive effect of hydrocortisone on systemic blood pressure.

Adrenal insufficiency in hypoxic ischemic injury could be due both to reduced perfusion of the adrenal gland and to a transient refractory state of the HPA axis. Hypoxic insult may affect the adrenal gland, leading to adrenal haemorrhage, impaired adrenal performance, low serum cortisol concentrations and an increased risk of heart dysfunction [42].

Hydrocortisone treatment is used frequently as a rescue therapy in asphyxiated newborns with vasopressor-resistant hypotension, and it appears to be a treatment option in several clinical review articles [43].

Steroid supplementation with hydrocortisone causes an increase in blood pressure soon after the start of treatment [44], but its administration should be considered carefully because of the risk of adverse neurodevelopmental effects (e.g., abnormal brain development) [45, 46].

Moreover, adrenal insufficiency is observed in critically ill neonates who are born at term, especially during the first week after delivery [47]. In this population, a strong improvement in hemodynamic parameters after hydrocortisone therapy was observed, unlike in neonates without evidence of adrenal failure.

The beneficial effect of hydrocortisone therapy has also been observed in premature newborns, with a reduction in the duration and/or amount of catecholamine administration [48–50].

We are not able to confirm whether the definition of CIRCI could be extended to neonatal age. Additionally, the use of glucocorticoids in critically ill neonates is controversial, so a specific characterization of this condition and of the most appropriate therapeutic approach should be provided by further studies.

### Major trauma and severe burns

CIRCI may be common in patients with major trauma, and it is associated with uncontrolled inflammation, vasopressor dependency and poor clinical outcomes.

Paediatric reports about this specific condition are not available, and we should refer only to adult protocols. Current guidelines suggest against steroid supplementation in this group of patients because of the absence of improvement in short-term mortality (Table 2).

Despite extensive research into CIRCI, there is little information about CIRCI in burn patients. It is well known that patients with major burn injury experience a severe systemic inflammatory response that puts them at risk for CIRCI [11].

This risk is particularly increased in patients with advanced age and a greater total body surface area (TBSA) burn, both of which are also independent risk factors for mortality after burn injury. It has been demonstrated that the early identification of the highest risk patients results in earlier initiation of physiologic steroid replacement and leads to a significant reduction in morbidity and mortality [51].

### Paediatric acute respiratory distress syndrome

Paediatric acute respiratory distress syndrome (PARDS) is another condition associated with CIRCI. The link between these conditions could be found in the setting of systemic dysregulated inflammation that characterizes acute respiratory distress syndrome pathophysiology [52].

Steroids are potentially useful in PARDS, but the last Paediatric Acute Lung Injury Consensus Conference guidelines (PALICC- 2) do not recommend corticosteroids as routine therapy [53]. There are several conditions for which steroid effects may be beneficial in patients with PARDS: treatment of a coexistent steroid-responsive disease; treatment of inflammation early to minimise lung damage and management of critical illness-related corticosteroid insufficiency [54].

The 2017 recommendations for the management of critical illness-related corticosteroid insufficiency do not analyse CIRCI in PARDS, but recommend methylprednisolone at a dose of 1 mg/kg/day in adults with ARDS and a PaO_2_/FiO_2_ < 200 within the first 6 days of illness. After day 6 of illness, they recommend increasing the dose to 2 mg/kg/day and a slow taper over at least 13 days (Table 2).

Although the use of corticosteroids in PARDS is not routinely recommended, the need for further study to identify the correct patient population, timing of administration and dosing regimen could also be useful for the management of the associated CIRCI condition.

## Conclusions

The diagnosis and management of CIRCI in paediatric age remain a challenge because of the absence of univocal indications supported by clinical trials and specific guidelines [6].

To the best of our knowledge, the present review is one of the few papers that provides a broad overview of the clinical presentation and management of paediatric CIRCI. Due to the insufficient evidence available on the diagnostic algorithm to be used in CIRCI in critically ill paediatric patients and pending new and specific paediatric guidelines, we must suggest that we often refer to guidelines for adult patients in our clinical practice. With the results of our literature search, we were able to confirm that many uncertainties remain, especially in the selection of patients for steroid treatment. For this reason, a reasonable approach could be to choose the children to be treated with steroids on the basis of a careful case-by-case evaluation until further definitive evidence is available.

Moreover, it should be considered that the indiscriminate use of steroid therapy in critically ill patients is not always justified and may cause harm, including impaired wound healing, hyperglycaemia, myopathy, and HPA suppression.

Further studies are needed to clarify all these points. There is also a need for a clinically useful ‘marker’ of glucocorticoid action in the context of tissue glucocorticoid resistance, which may be related to alteration in the function of intracellular glucocorticoid receptors.

## Data Availability

All studies and data analysed during this study are included in this article. Further enquiries can be directed to the corresponding author.

## Abbreviations

CIRCI: Critical illness-related corticosteroid insufficiency
HPA: Hypothalamic–pituitary–adrenal
ICU: Intensive Care Unit
CRH: Corticotropin-releasing hormone
ACTH: Adrenocorticotropin hormone
RAI: Relative Adrenal Insufficiency
AAI: Absolute Adrenal Insufficiency
TNF-α: Tumor necrosis factor-α
